# Impact of community-based interventions for the prevention and control of malaria on intervention coverage and health outcomes for the prevention and control of malaria

**DOI:** 10.1186/2049-9957-3-25

**Published:** 2014-08-01

**Authors:** Rehana A Salam, Jai K Das, Zohra S Lassi, Zulfiqar A Bhutta

**Affiliations:** 1 Division of Women and Child Health, The Aga Khan University, Karachi, Pakistan; 2 Center of Excellence in Women & Child Health, The Aga Khan University, Karachi, Pakistan; 3 Center for Global Child Health Hospital for Sick Children, Toronto, Canada

**Keywords:** Malaria, Community-based interventions, Malarial control, Malaria treatment

## Abstract

In this paper, we aim to evaluate the effectiveness of community-based interventions (CBIs) for the prevention and management of malaria. We conducted a systematic review and identified 42 studies for inclusion. Twenty-five of the included studies evaluated the impact of the community-based distribution of insecticide-treated nets (ITNs), indoor residual spraying (IRS), or impregnated bed sheets; 14 studies evaluated intermittent preventive therapy (IPT) delivered in community settings; two studies focused on community-based education for malaria prevention; and one study evaluated environmental management through drain cleaning.

Our analysis suggests that, overall, the community-based delivery of interventions to prevent and control malaria resulted in a significant increase in ITNs ownership (RR: 2.16, 95% CI: 1.86, 2.52) and usage (RR: 1.77, 95% CI: 1.48, 2.11). However, usage of ITNs was limited to two-thirds of the population who owned them. Community-based strategies also led to a significant decrease in parasitemia (RR: 0.56, 95% CI: 0.42, 0.74), malaria prevalence (RR: 0.46, 95% CI: 0.29, 0.73), malaria incidence (RR: 0.70, 95% CI: 0.54, 0.90), and anemia prevalence (RR: 0.79, 95% CI: 0.64, 0.97). We found a non-significant impact on splenomegaly, birth outcomes (low birth weight, prematurity, stillbirth/miscarriage), anthropometric measures (stunting, wasting, and underweight), and mortality (all-cause and malaria-specific). The subgroup analysis suggested that community-based distribution of ITNs, impregnated bed sheets and IRS, and IPT are effective strategies. Qualitative synthesis suggests that high coverage could be achieved at a lower cost with the integration of CBIs with existing antenatal care and immunization campaigns. Community-based delivery of interventions to prevent and control malaria are effective strategies to improve coverage and access and reduce malaria burden, however, efforts should also be concerted to prevent over diagnosis and drug resistance.

## Multilingual abstracts

Please see Additional file 1 for translations of the abstract into the six official working languages of the United Nations.

## Introduction

Malaria is a parasitic infection spread by the female *Anopheles* mosquito and is responsible for 660,000 deaths globally and over 219 million cases of infection annually [1]. High-income countries (HICs) have been able to eliminate malaria, while many parts of low- and middle-income countries (LMICs) are still struggling to deal with malaria and vector control [1]. In 2012, out of the 104 malaria endemic countries, 79 countries are in the malaria control phase, ten are in the pre-elimination phase, ten are in the elimination phase, and five are focusing on the prevention of re-introduction [1]. A number of factors account for the existing malaria burden in developing countries including climate change, infrastructure, emerging drug and insecticide resistance, massive population and demographic shifts, and costs of containment and therapy. In Sub-Saharan Africa, the wide-scale implementation of insecticide-treated bed nets (ITNs) has been one of the main strategies to reduce malaria morbidity and mortality [2].

There are existing interventions for malaria prevention, which include indoor residual spraying (IRS), ITNs, intermittent preventive therapy (IPT), presumptive treatment, and education. Previously, malarial chemoprophylaxis with chloroquine (CQ) was generally recommended for pregnant women in malaria endemic regions, however, with the recent increase in *Plasmodium falciparum* resistance to CQ [3-5], the World Health Organization (WHO) recommends the use of sulfadoxine-pyrimethamine (SP) for the intermittent preventive treatment in pregnancy (IPTp). In this paper, we aim to evaluate the effectiveness of community-based interventions (CBIs) and their impact on the prevention and control of malaria.

## Methods

We systematically reviewed literature published before May 2013 to identify studies evaluating the effectiveness of the community-based delivery of interventions for the prevention and control of malaria as outlined in our conceptual framework [6]. We included randomized controlled trials (RCTs), quasi-experimental trials, and before-and-after studies, in which the interventions for the prevention and control of malaria were delivered within community settings and the reported outcomes were relevant. A comprehensive search strategy was developed using appropriate keywords, medical subject headings (MeSH), and free text terms. Searches were conducted in PubMed, Cochrane Libraries, Embase, and WHO Regional Databases. Studies that met the inclusion criteria were selected and double data abstracted on a standardized abstraction sheet. We excluded studies in which interventions were delivered in antenatal or immunization clinics, or if social marketing strategies in combination with facility-based interventions were evaluated. Studies were also excluded if the interventions were given to the displaced population or if the efficacy/effectiveness of a particular brand of bed nets, drugs, or diagnostic tools was evaluated. Studies reporting only entomological indices and parasite prevalence were also excluded. Quality assessment of the included RCTs was done using the Cochrane risk of bias assessment tool [7]. The outcomes of interest are outlined in Table 1. We conducted a meta-analysis for individual studies using the software Review Manager 5.1. Pooled statistics were reported as the relative risk (RR) for categorical variables and standard mean difference (SMD) for continuous variables between the experimental and control groups with 95% confidence intervals (CIs). We also attempted to qualitatively synthesize the findings reported in the included studies for other pragmatic parameters identified in our conceptual framework including intervention coverage, challenges/barriers, enabling factors, aspects related to integrated delivery, monitoring and evaluations and equity. The detailed methodology is described in a separate paper [6].

**Table 1:** Outcomes analyzed

**Outcomes**	**Outcomes analyzed**
**Morbidity**	Parasitemia
	Malaria incidence
	Malaria prevalence
	Splenomegaly
**Anthropometry**	Weight
	Height
	Stunting
	Wasting
	Underweight
**Hematologic**	Prevalence of anemia
	Mean hemoglobin
**Birth Outcomes**	Birth weight
	Prematurity
	Low birth weight (LBW)
	Stillbirth/miscarriage
**Mortality**	All-cause mortality
	Malaria specific mortality
**Coverage**	ITNs ownership
	ITNs usage (sleeping under bed nets)

## Review

**Figure 1:**
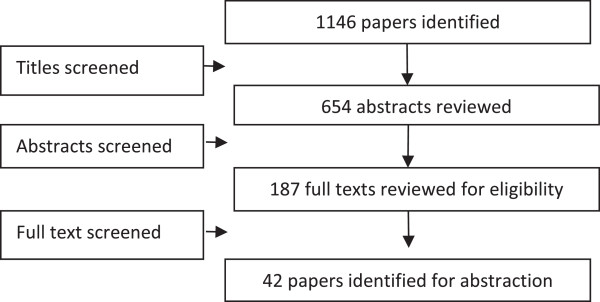
Search flow diagram.

We identified 1,146 titles from the search conducted in all databases. After screening the titles and abstracts, 187 full texts were reviewed, of which 42 studies (17 RCTs, 10 quasi-experimental trials, 13 before-and-after studies, and two case control studies) were included in the review (see Figure 1). The characteristics of the included studies are summarized in Table 2. Of the 42 studies, four studies could not be included in the meta-analysis as these did not report poolable data [8-11], while for studies reporting multiple evaluations of a single intervention, we pooled the results from the last reported survey [12,13]. From the 17 RCTs included in this review, randomization was adequate in six studies, allocation was concealed in six studies, and adequate sequence generation was done in four studies. None of the studies blinded participants due to the nature of the interventions, while all studies provided insufficient information on selective reporting which limited us from making any judgment (see Table 3).

**Table 2:** Characteristics of the included studies

**Study**	**Study design**	**Country**	**Intervention**	**Target population**	**Integrated/Non-integrated**
Abdulla 2001 [14]	Pre-post	Tanzania	Social marketing about bed nets and ITNs through public and private outlets and door-to-door distribution	Interventions for the general population and outcomes assessed in children under 2 years of age	Non-integrated
Ahorlu 2009 [13]	Pre-post	Ghana	Home delivered IPTc comprising of a single dose of amodiaquine (AQ) + artesunate (AS) over three days along with treatment of febrile illness	Children 6–60 months of age	Non-integrated
Ahorlu 2011 [12]	Pre-post	Ghana	Home delivered IPTc comprising of a single dose of AQ + AS over three days along with treatment of febrile illness	Children 6–60 months of age	Non-integrated
Ayi 2010 [15]	Quasi-experimental	Ghana	School-based malaria education delivered by teachers	School children in grades 3–5	Non-integrated, school- based
Bojang 2011 [16]	cRCT	Gambia	IPTc delivered by village health workers (VHW) comprising of a single dose of sulfadoxine (SP) + three doses of AQ versus delivery through reproductive and child health clinics	Children < 6 years	Non-integrated
Castro 2009 [17]	Quasi-experimental	Tanzania	Environmental management through drain cleaning versus use of larvicide versus no intervention	General population	Non-integrated
D’Alessandro 1995 [16]	cRCT	Gambia	Impregnated bed nets distribution	General population	Integrated with PHC and delivered through TBA
D’Alessandro 1995 [18]	cRCT	Gambia	Impregnated bed nets distribution	Children 1–4 years	Integrated with PHC and delivered through TBA
D’Alessandro 1996 [19]	cRCT	Gambia	Impregnated bed nets distribution	Pregnant women	Integrated with PHC and delivered through TBA
D’Alessandro 1997 [8]	Case control	Gambia	Impregnated bed nets distribution	Children 1–9 years of age	Integrated with PHC and delivered through TBA
Dapeng 1996 [20]	Pre-post	China	Insecticide spraying + ITNs	General population	Non-integrated
Delacollette 1996 [21]	Quasi-experimental	Zaire	Educational messages + presumptive treatment with chloroquine (CQ) through CHWs versus routine treatment in a health facility	General population	Non-integrated
Eriksen 2010 [18]	cRCT	Tanzania	Training of health workers and women leaders + presumptive malaria treatment with single dose SP versus routine facility care	Children < 5 years	Non-integrated
Ter Kuile 2003 [22]	cRCT	Kenya	ITNs distribution versus no distribution	Children < 36 months	Non-integrated
Gies 2008 [23]	Quasi-experimental	Burkina Faso	Community based promotion + IPTp with SP versus only IPTp with SP versus CQ	Pregnant women	Integrated with ANC
Grabowski 2005 [24]	Pre-post	Ghana	ITNs distribution	Children < 5 years	Integrated with measles campaign
Grabowski 2005 [25]	Pre-post	Zambia	ITNs distribution	Children < 5 years	Integrated with measles campaign
Greenwood 1989 [26]	RCT	Gambia	IPTp with Maloprim fortnightly delivered through TBA	Pregnant women	Integrated with ANC delivered through TBA
Hawley 2003 [10]	RCT	Ghana	Community education + ITNs distribution	General population	Non-integrated
Hightower 2010 [27]	Pre-post	Kenya	Free distribution	Pregnant women and children < 5 years	Integrated with measles campaign
Kidane and Morrow, 2000 [28]	RCT	Ethiopia	Mothers trained to provide presumptive treatment to children with CQ	Children < 5 years	Non-integrated
Kolaczinski 2010 [29]	Quasi-experimental	Uganda	ITNs distribution campaign	Pregnant women and children < 5 years	Non-integrated
Krezanoski 2010 [30]	cRCT	Madagascar	Distribution of redeemable coupons for ITNs	General population	Non-integrated
Kuile 2003 [31]	RCT	Kenya	Distribution of ITNs versus no nets	Intervention on general population while outcomes were assessed on children	Non-integrated
Kweku 2009 [32]	cRCT	Ghana	IPTc comprising of three doses of AQ + SP delivered by community volunteers versus delivery at outpatient facilities or EPI clinics	Children < 5 years	Non-integrated
Macintyre 2003 [33]	RCT	Kenya	Use of impregnated bed sheets	General population	Non-integrated
Mbonye 2008 [34]	Quasi-experimental	Uganda	IPTp with two doses of SP delivered through TBA shop vendors, community reproductive health workers and APMs	Pregnant women	Integrated with ANC through TBAs
Mbonye 2008 [35]	Quasi-experimental	Uganda	IPTp with two doses of SP delivered through TBA shop vendors, community reproductive health workers and APMs	Pregnant women	Integrated with ANC through TBAs
Msyamboza 2008 [36]	Quasi-experimental	Malawi	IPTp comprising threw doses of SP delivered through SP versus facility-based care	Pregnant women	Integrated with ANC
Noor 2007 [37]	Pre-post	Kenya	ITNs delivered via the commercial sector versus ANC clinics versus mass distribution	General population	Non-integrated
Okabayashi 2006 [38]	Pre-post	Thailand	School-based teacher training and manual formulation for children, school lectures, outdoor activities, and community awareness about malaria prevention	School children from grades 3–5	School-based, non-integrated
Okeibunor 2011 [39]	Quasi-experimental	Nigeria	Distribution of IPTp (SP) + ITNs through community volunteers	Pregnant women	Non-integrated
Rhee 2005 [40]	Quasi-experimental	Mali	Community education + net impregnation services versus net impregnation alone	General population	Non-integrated
Schellenberg 2001 [41]	Pre-post	Tanzania	Social marketing of ITNs + insecticides for ITNs through community shop keepers, religious leaders, and health workers with community sensitization	General population	Non-integrated
Sharma 2009 [42]	RCT	India	Community group meeting for ITNs use and mass awareness + olyset nets versus untreated nets versus no nets	General population	Non-integrated
Skarbinski 2007 [43]	Pre-post	Tanzania	ITNs distribution with a child health campaign including measles, vitamin A, and deworming	Children < 5 years	Integrated with child health campaign
Staedke 2009 [44]	RCT	Uganda	Home-based presumptive treatment with artemether/lumefantrine versus clinic-based routine care	Children 1–6 years	Non-integrated
Tagbor 2011 [45]	RCT	Ghana	Presumptive malaria treatment by community drug distributors (home based management) + IPTc with AS and AQ	Children < 5 years	Non-integrated
Terlouw 2010 [46]	Pre-post	Togo	ITNs distribution with a child health campaign	Children < 5 years	Integrated with child health campaign
Thang 2009 [47]	cRCT	Vietnam	Distribution of long-lasting insecticide-treated hammocks	General population	Non-integrated
Thwing 2008 [48]	Pre-post	Niger	ITNs distribution with polio immunization	Children < 5 years	Integrated with polio immunization
Wolkon 2010 [49]	Pre-post	Togo	ITNs distribution with a deworming and vaccine campaign during child health week	Children + outcomes in general population	Integrated with child health days

**Table 3:** Quality assessment of the included RCTs

**Study**	**Randomization**	**Sequence generation**	**Allocation concealment**	**Blinding of participants**	**Blinding of assessors**	**Selective reporting**
Bojang 2011 [16]	Done	Done	Not done	Not done	Not clear	No
D’Alessandro 1995 [50]	Not clear	Not clear	Not done	Not done	Not done	No
D’Alessandro 1995 [9]	Not clear	Not clear	Not done	Not done	Not done	No
D’Allessandro 1996 [19]	Not clear	Not clear	Not done	Not done	Not done	No
Eriksen 2010 [18]	Done	Done	Not done	Not done	Not clear	No
Ter Kuile 2003 [31]	Done (Not clear)	Not clear	Not done	Not done	Not clear	No
Greenwood 1989 [26]	Not clear	Not clear	Not done	Not done	Not clear	No
Hawley 2003 [10]	Done (Not clear)	Not clear	Not done	Not done	Not clear	No
Kidane 2000 [28]	Done (Not clear)	Not clear	Not done	Not done	Not clear	No
Krezonoski 2010 [30]	Done	Not done	Not done	Not done	Not clear	No
Kuile 2003 [22]	Done (Not clear)	Not clear	Not done	Not done	Not clear	No
Kweku 2009 [32]	Done	Not done	Not done	Not done	Not clear	No
Macintyre 2003 [33]	Done (Not clear)	Not clear	Not done	Not done	Not clear	No
Sharma 2009 [42]	Done (Not clear)	Not clear	Not done	Not done	Not clear	No
Staedke 2009 [44]	Done	Done	Not done	Not done	Not clear	No
Tagbor 2011 [45]	Done (Not clear)	Not clear	Not done	Not done	Not clear	No
Thang 2009 [47]	Done	Done	Not done	Not done	Not clear	No

Twenty-five of the included studies evaluated the impact of the community-based distribution of ITNs or impregnated bed sheets, 14 studies evaluated IPT delivered in community settings, two studies focused on community-based education for malaria prevention, and one study evaluated environmental management through drain cleaning. Community education on malaria preventive measures was one of the components of interventions in most of the studies. All the studies were conducted in African countries except for three studies, one each from China, India, and Thailand. Interventions were non-integrated in 23 [10,12-14,16-18,20-22,28-33,37,39-42,45,47] of the studies, while in 17 [8,9,19,23-27,34-36,43,44,46,48-50] studies intervention was integrated with routine community-based antenatal care (ANC), primary healthcare (PHC), child health days or measles, and polio campaigns. The primary comparison was between the community-based delivery strategy versus routine or facility-based care. We also attempted to conduct a subgroup analysis to determine the relative effectiveness of integrated and non-integrated delivery strategies, according to the type of intervention and whether the evidence was from RCT/quasi-experimental studies or pre-post studies, where possible. The results are summarized in Tables 4 and 5.

**Table 4:** Results for the overall and subgroup analysis according to the type of study and treatment

**Outcomes**	**Estimates (95% CI)**				
	**Combined**	**RCTs**	**Pre-post studies**	**Community-based, non-integrated delivery**	**Community-based, integrated delivery**
**Coverage outcomes**					
**ITNs ownership**	**2.16 [1.86, 2.52]**, 15 datasets 14 studies	0.97 [0.94, 1.00], 5 datasets 4 studies	**3.71 [2.62, 5.27]**, 10 datasets 10 studies	**1.24 [1.11, 1.39]**, 8 datasets 7 studies	**5.05 [2.59, 9.86]**, 7 datasets 7 studies
**ITNs usage**	**1.77 [1.48, 2.11]**, 16 datasets 15 studies	1.03 [0.91, 1.15], 9 datasets 8 studies	**3.75 [2.35, 5.99]**, 7 datasets 7 studies	**1.18 [1.03, 1.34]**, 10 datasets 9 studies	**6.97 [3.10, 15.69]**, 6 datasets 6 studies
**Morbidity outcomes**					
**Parasitemia**	**0.56 [0.42, 0.74]**, 11 datasets 10 studies	**0.64 [0.48, 0.85]**, 9 datasets 8 studies	0.15 [0.01, 2.56], 2 datasets 2 studies	**0.39 [0.24, 0.64]**, 5 datasets 5 studies	**0.72 [0.53, 0.99]**, 6 datasets 5 studies
**Malaria prevalence**	**0.46 [0.29, 0.73]**, 10 datasets 9 studies	**0.52 [0.32, 0.85]**, 8 datasets 7 studies	0.29 [0.05, 1.78], 2 datasets 2 studies	**0.42 [0.25, 0.69]**, 9 datasets 8 studies	0.29 [0.05, 1.78], 1 dataset 1 study
**Malaria incidence**	**0.70 [0.54, 0.90]**, 5 datasets 5 studies	**0.70 [0.54, 0.90]**, 5 datasets 5 studies	No studies	**0.70 [0.54, 0.90]**, 5 datasets 5 studies	No studies
**Splenomegaly**	0.75 [0.52, 1.06], 4 datasets 4 studies	0.91 [0.74, 1.11], 3 datasets 3 studies	**0.57 [0.49, 0.65]**, 1 dataset 1 study	**0.57 [0.50, 0.65]**, 2 datasets 2 studies	0.92 [0.75, 1.13], 2 datasets 2 studies
**Hematologic markers**					
**Anemia prevalence**	0.79 [0.64, 0.97], 10 datasets 9 studies	0.91 [0.75, 1.11], 9 datasets 8 studies	**0.53 [0.43, 0.65]**, 2 datasets 2 studies	**0.71 [0.53, 0.97]**, 6 datasets 6 studies	0.98 [0.71, 1.35], 5 datasets 4 studies
**Mean Hb**	1.85 [-0.85, 4.55], 5 studies 5 studies	-0.03 [-0.40, 0.34], 4 datasets 4 studies	**9.00 [8.80, 9.20]**, 1 dataset 1 study	2.22 [-0.77, 5.22], 4 datasets 4 studies	0.20 [-1.80, 2.20], 1 dataset 1 study
**Birth outcomes**					
**Birth weight**	22.68 [-54.26, 99.62], 3 datasets 3 studies	22.68 [-54.26, 99.62], 3 datasets 3 studies	No studies	No studies	22.68 [-54.26, 99.62], 3 datasets 3 studies
**LBW**	0.95 [0.63, 1.44], 4 datasets 3 studies	0.95 [0.63, 1.44], 4 datasets 3 studies	No studies	No studies	0.95 [0.63, 1.44], 4 datasets 3 studies
**Prematurity**	0.42 [0.13, 1.36], 1 dataset 1 study	0.42 [0.13, 1.36], 1 dataset 1 study	No studies	No studies	0.42 [0.13, 1.36], 1 dataset 1 study
**Stillbirth/miscarriage**	1.23 [0.90, 1.69], 2 datasets 1 study	1.23 [0.90, 1.69], 2 datasets 1 study	No studies	No studies	1.23 [0.90, 1.69], 2 datasets 1 study
**Anthropometry outcomes**					
**Weight**	-0.02 [-0.28, 0.24], 2 datasets 2 studies	0.00 [-0.28, 0.28], 1 dataset 1 study	-0.10 [-0.72, 0.52], 1 dataset 1 study	-0.02 [-0.28, 0.24], 2 datasets 2 studies	No studies
**Stunting**	1.11 [0.86, 1.42], 1 dataset 1 study	1.11 [0.86, 1.42], 1 dataset 1 study	No studies	1.11 [0.86, 1.42], 1 dataset 1 study	No studies
**Wasting**	0.87 [0.67, 1.13], 1 dataset 1 study	0.87 [0.67, 1.13], 1 dataset 1 study	No studies	0.87 [0.67, 1.13], 1 dataset 1 study	No studies
**Underweight**	0.94 [0.78, 1.14], 1 dataset 1 study	0.94 [0.78, 1.14], 1 dataset 1 study	No studies	0.94 [0.78, 1.14], 1 dataset 1 study	No studies
**Mortality**					
**All-cause mortality**	0.81 [0.56, 1.15], 3 datasets 3 studies	0.81 [0.56, 1.15], 3 datasets 3 studies	No studies	0.89 [0.37, 2.15], 1 dataset 1 study	**0.79 [0.64, 0.96]**, 1 dataset 1 study
**Malaria specific mortality**	0.54 [0.21, 1.40], 2 datasets 2 studies	0.54 [0.21, 1.40], 2 datasets 2 studies	No studies	**0.33 [0.20, 0.55]**, 1 dataset 1 study	0.86 [0.62, 1.19], 1 dataset 1 study

**Table 5:** Summary of evidence according to the type of intervention

**Outcomes**	**ITNs, bed sheets, and IRS**	**IPT**	**Community education and cleanliness campaigns**
ITNs ownership	**2.28 [1.95, 2.67]**		0.99 [0.76, 1.28]
ITNs usage	**2.49 [1.90, 3.27]**	1.07 [0.59, 1.94]	1.02 [0.96, 1.09]
Parasitemia	**0.58 [0.36, 0.94]**	**0.54 [0.37, 0.81]**	
Malaria prevalence	**0.42 [0.25, 0.70]**	0.45 [0.14, 1.47]	0.53 [0.11, 2.59]
Malaria incidence	0.74 [0.53, 1.04]	0.50 [0.22, 1.14]	
Splenomegaly	0.72 [0.44, 1.17]	0.82 [0.52, 1.32]	
Anemia prevalence	**0.49 [0.38, 0.62]**	0.90 [0.76, 1.07]	
Mean Hb	**9.00 [8.80, 9.20]**	-0.03 [-0.40, 0.34]	
Weight		-0.02 [-0.28, 0.24]	
Stunting		1.11 [0.86, 1.42]	
Wasting		0.87 [0.67, 1.13]	
Underweight		0.94 [0.78, 1.14]	
All-cause mortality	**0.79 [0.64, 0.96]**	0.89 [0.37, 2.15]	
Malaria specific mortality	0.86 [0.62, 1.19]	**0.33 [0.20, 0.55]**	

ITNs: Insecticide-treated nets, IRS: Indoor residual spraying, IPT: Intermittent preventive therapy.

*Estimates in bold suggests significant impact.

### Quantitative synthesis

**Figure 2:**
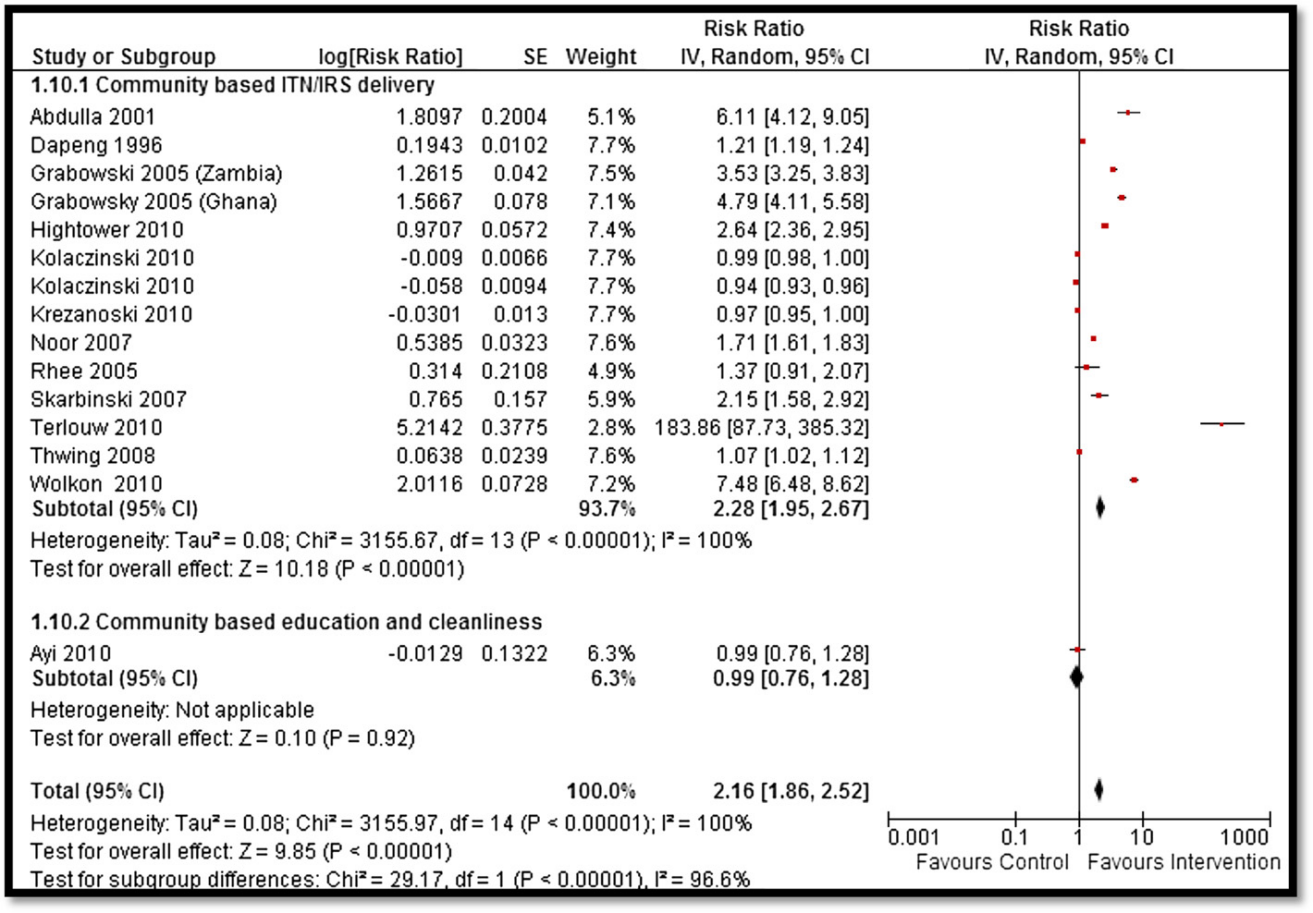
Forest plot for the impact of CBIs on ITNs ownership.

**Figure 3:**
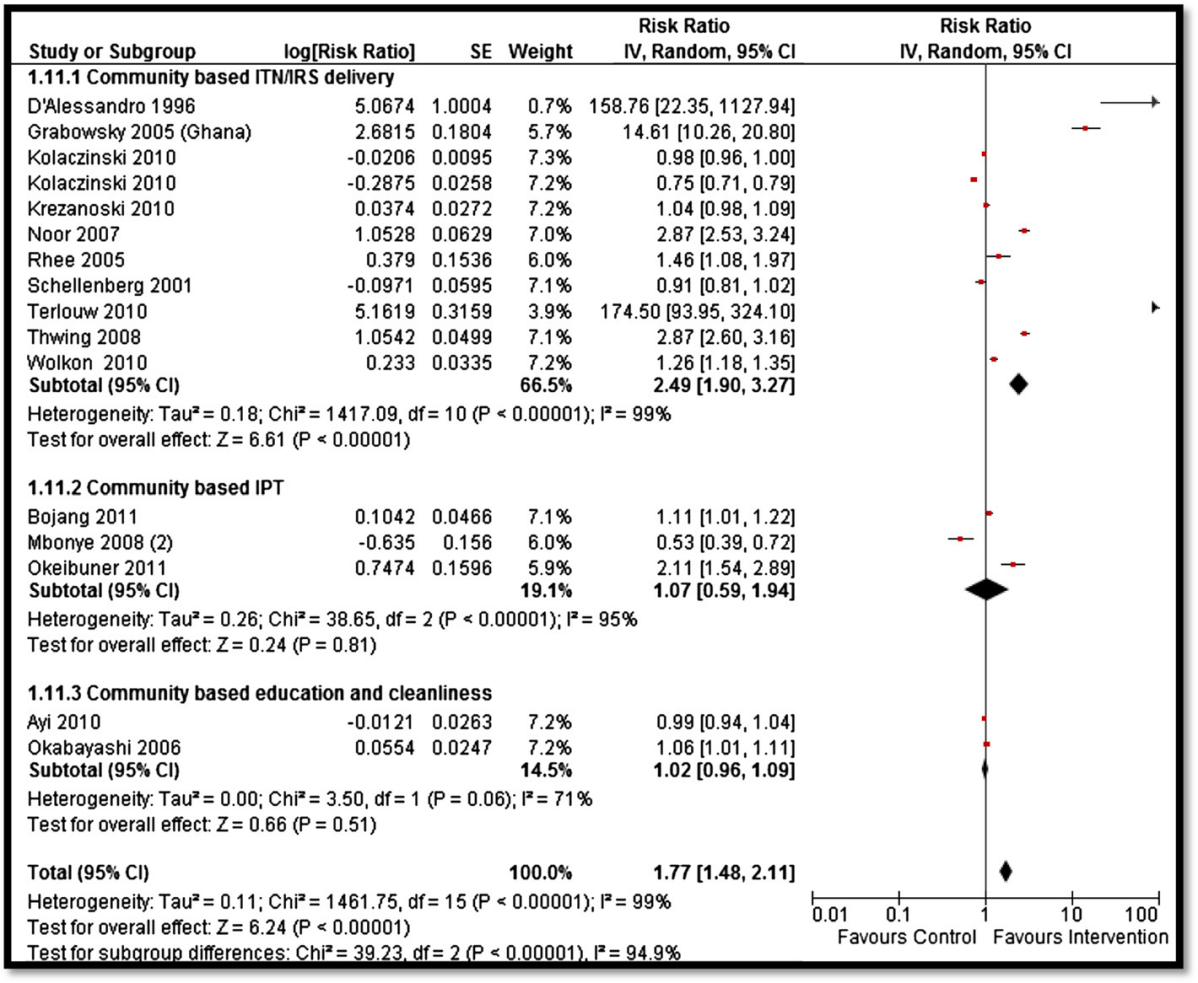
Forest plot for the impact of CBIs on ITNs usage.

**Figure 4:**
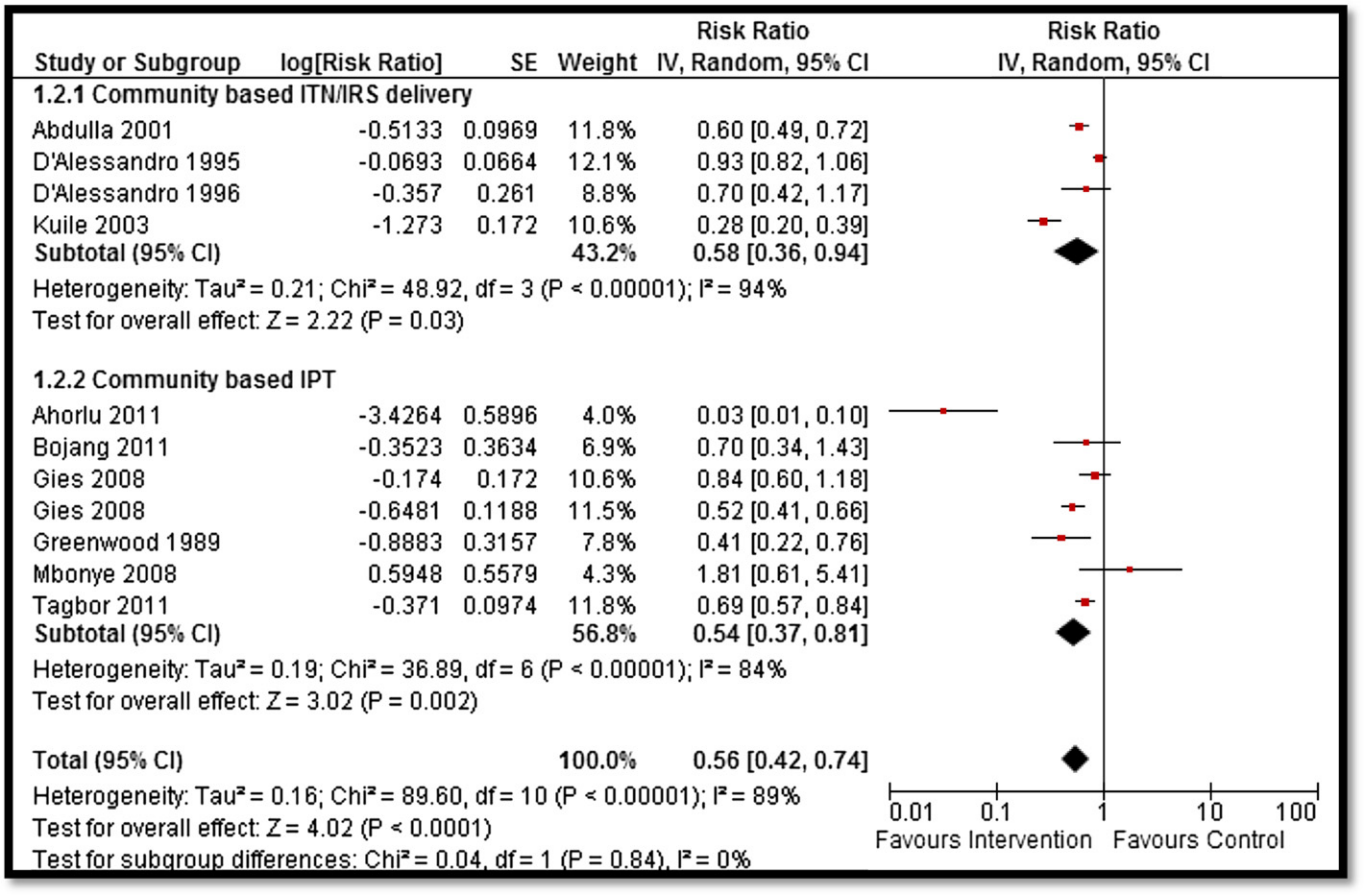
Forest plot for the impact of CBIs on parasitemia.

**Figure 5:**
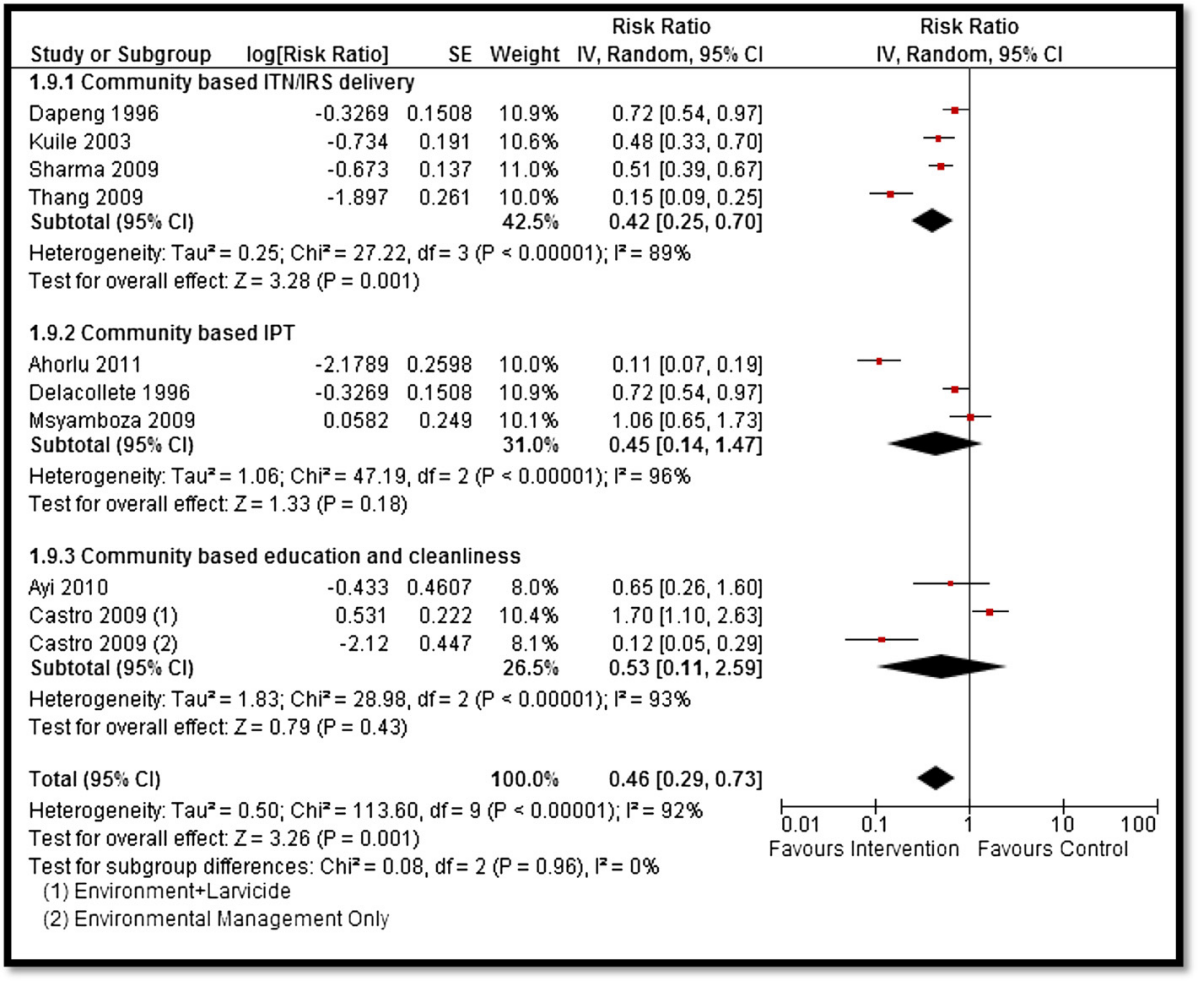
Forest plot for the impact of CBIs on malaria prevalence.

**Figure 6:**
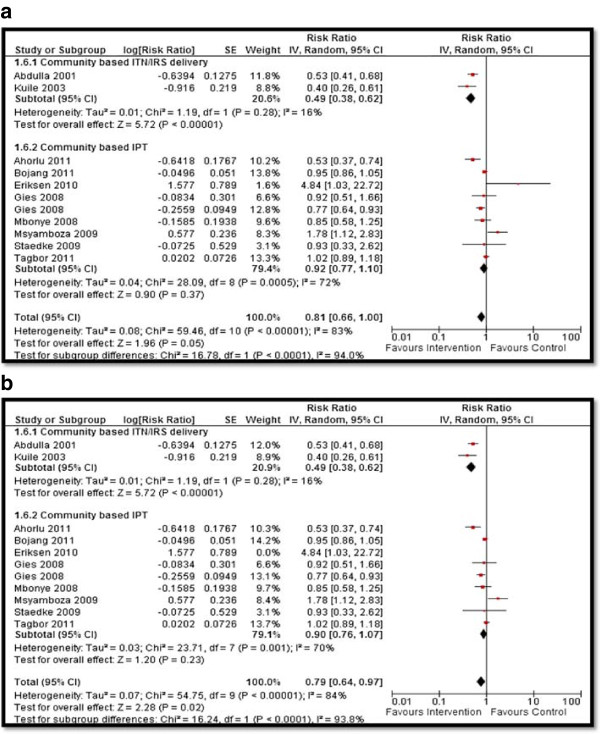
Forest plot for the impact of CBIs on anemia (a) with all studies included (b) after sensitivity analysis.

Table 4 depicts the impact of the overall community-based delivery of interventions and the subgroup analysis according to the type of study and intervention. Overall, community-based delivery of interventions to prevent and control malaria resulted in a significantly higher ownership (RR: 2.16, 95% CI: 1.86, 2.52) and usage (RR: 1.77, 95% CI: 1.48, 2.11) of ITNs in the intervention group as compared to the control group (see Figures 2 and 3). Ownership was defined as households having at least one net at the time of the survey, while usage was defined as having slept under an ITN the previous night or having an ITN hanging over the bed. The usage rate of ITNs among people who owned an ITN was around 66%. Community-based delivery strategy was also associated with significantly lower malaria incidence (RR: 0.70, 95% CI: 0.54, 0.90), parasitemia (RR: 0.56, 95% CI: 0.42, 0.74), and malaria prevalence (RR: 0.46, 95% CI: 0.29, 0.73) in the intervention group (see Figures 4 and 5). Anemia prevalence also reduced significantly (RR: 0.79, 95% CI: 0.64, 0.97) with sensitivity analysis after removing Eriksen 2010 (which reported concurrent interventions in both groups due to the introduction of a national campaign during the study period) (see Figure 6). We found non-significant impact on mean hemoglobin, splenomegaly, birth outcomes (low birth weight [LBW], prematurity, stillbirth/miscarriage), anthropometric measures (stunting, wasting, and underweight), and mortality (all-cause and malaria-specific). These findings are based on limited number of studies pooled with a high level of heterogeneity and hence should be interpreted with caution. The subgroup analysis for integrated and non-integrated delivery showed significant impacts on all outcome indicators except for malaria prevalence and splenomegaly which was non-significant for the integrated delivery subgroup, though this is based on a limited number of studies.

Table 5 summarizes the evidence from the subgroup analysis according to the type of intervention. The community-based distribution of ITNs, impregnated bed sheets, and IRS resulted in significantly higher ITNs ownership (RR: 2.28, 95% CI: 1.95, 2.67), ITNs usage (RR: 2.49, 95% CI: 1.90, 3.27), and mean hemoglobin levels (SMD: 9.00, 95% CI: 8.80, 9.20). It also led to a significant reduction in parasitemia (RR: 0.58, 95% CI: 0.36, 0.94), malaria prevalence (RR: 0.42, 95% CI: 0.25, 0.70), anemia prevalence (RR: 0.49, 95% CI: 0.38, 0.62), and all-cause mortality (RR: 0.79, 95% CI: 0.64, 0.96). The community-based delivery of IPT significantly reduced parasitemia (RR: 0.54, 95% CI: 0.37, 0.81) and malaria-specific mortality (RR: 0.33, 95% CI: 0.20, 0.55). Community education and cleanliness campaigns alone did not show any significant impact on the outcomes measured.

### Qualitative synthesis

Interventions delivered in community setups reported great potential to improve coverage, access, and adherence to ITNs and IPT, as these were delivered via community volunteers who were easily accessible and trusted resource persons and who could make regular home visits and follow up with their patients [34]. Delivering intermittent preventive therapy during childhood (IPTc) through community health workers (CHWs) has shown several advantages as CHWs are community residents and can not only deliver effective and timely treatment, but also remind mothers/guardians if they forget to attend treatment. Thus, operationally, delivery using CHWs was less restrictive and more convenient for parents and guardians [16]. Furthermore, CHWs also contributed to improving recognition and referral of seriously ill patients, provided advice on hygiene and nutrition, and encouraged women to attend antenatal clinics and to immunize their children [12,13,16]. However, whether to give incentives to CHWs to encourage sustainability is still debated as some mass drug delivery systems have been successful without incentives whilst others have employed financial incentives of some kind [16]. Studies suggest that financial payment and a strengthened drug supply may contribute to program success, however, incentives must reach the CHWs in a timely and efficient manner to avoid demotivation [16]. Besides incentivizing, CHWs require proper training, facilitation, and linkage with health units coupled with the provision of reference manual for easy and quick referencing to deliver timely treatment [12,13,34].

The integration of CBIs for malaria with existing ANC and immunization campaigns is reportedly more feasible and acceptable, and has reported improved coverage of IPTp to pregnant women [36]. The integration of malaria control programs with such promotional campaigns has resulted in a major increase in treatment coverage and ITNs distribution at a very low cost [23,24,36,43]. Several features of ITNs distribution and mass measles vaccination campaigns favor the sustainability of an integrated approach. These include high coverage and low cost, as well as the fact that ITNs require replacing at the same intervals as measles vaccination campaigns take place [24,27]. However, effective integration requires careful planning to ensure that each component of the package is not jeopardized by the other [37]. Some of the other strategies shown to achieve high and equitable coverage include mass free distribution and social marketing [10,14,24,27,30,37,41].

Factors enabling the delivery of CBIs mainly involved community empowerment, intensive social mobilization, and education [24]. The implementation of environmental management activities at the community level requires empowering local residents, developing a sense of ownership, and improving environmental responsibility among the population [17]. Providing incentives, social marketing, and subsidization of the costs of ITNs have also been reported as powerful tools, especially for programs seeking coverage for vulnerable groups such as children and pregnant women [30]. However, household ownership should be followed up to ensure usage. Studies have reported that barriers to ITNs use were not only the costs and access to ITNs, but also fear about insecticides and a lack of knowledge about malaria and ITNs [27]. Other reasons for not using an ITN included discomfort, problems with hanging up the nets and lack of space, low awareness of its need, and seasonal variations in use [51]. Community education together with other interventions for malaria prevention can have a substantial impact on increasing the usage of ITNs [15]. Educational interventions based on lectures and theoretical case studies without any follow-up training have been shown to be less effective than multifaceted interventions involving other strategies [18]. School-based interventions involving school teachers delivering educational messages through activities such as role-playing, poetry recitals, slogan chanting, song composition, and dramatization have reportedly been acceptable and effective [15], however, these require a well-established school health system [15].

## Discussion

Our review findings suggest that community-based delivery of interventions to prevent and control malaria is effective in improving ITNs ownership and usage, and reducing malaria incidence, parasitemia, malaria prevalence and anemia. However, this strategy did not have a statistically significant impact on birth outcomes, anthropometric measures, all-cause mortality, and malaria-specific mortality. These non-significant findings could be attributed to the concurrent national level malaria control measures being implemented in many of the African countries which could have led to more effective malaria control measures in both the intervention and control clusters since most of the studies included in our review are from Africa. Furthermore, the non-significant findings could also be attributable to the limited number of studies included for some of the outcomes (see Table 4). Our findings suggest a decrease in anemia prevalence, however, the mean hemoglobin remained non-significant. This could be due to various other causes of anemia coexisting with malaria in the study population (including infections, infestation, and malnutrition), the limited number of studies included, and a high level of heterogeneity in the pooled analysis. Such interactions and effect modifications should be considered when interpreting these findings.

Evidence from the subgroup analysis according to the type of interventions suggests that community-based distribution of ITNs, impregnated bed sheets, and IRS can effectively increase ITNs ownership, usage and mean hemoglobin levels, and effectively reduce parasitemia, malaria prevalence, anemia prevalence and all-cause mortality. The community-based delivery of IPT was found to be effective in reducing parasitemia and malaria-specific mortality, while community education and cleanliness campaigns alone did not show any improvement in the outcomes measured. The non-significant impact of the various types of interventions could also be attributable to the limited number of studies included for some of the outcomes in the subgroup analysis and a high level of heterogeneity. We did not find any conclusive evidence on the relative effectiveness of integrated and non-integrated delivery strategies from our quantitative synthesis due to limited data in each subgroup. However, the qualitative synthesis of the data from the included studies suggests that high coverage could be achieved at a lower cost with the integration of CBIs with existing ANC and immunization campaigns. There is a need for large-scale effectiveness trials to explore the contextual parameters associated with scaling-up CBIs for malaria prevention and management.

Merely half of the countries with ongoing malaria transmission are on track to meet the World Health Assembly’s (WHA’s) target of 75% reduction in malaria cases by 2015. The WHO recommends prompt and effective treatment with artemisinin-based combination therapies, use of ITNs by people at risk, and IRS with insecticide as the key interventions to control malaria. The past decade has witnessed some tremendous expansion in the implementation of malaria control programs with an increase in international disbursements from less than USD $100 million in 2000 to USD $1.71 billion in 2010, mainly targeting the African Region [1]. Treatment of malaria among children and pregnant women has also expanded coverage in many countries recently. However, millions of people still continue to lack access to preventive therapies, diagnostic testing, and quality-assured treatment with an emerging challenge being the resistance to artemisinins and mosquito resistance to insecticides. Some of the other reported factors affecting the delivery, access, and use of interventions to prevent malaria include unclear policy and guidance; general healthcare system issues, such as stock outs and user fees; health facility issues stemming from poor organization and leading to poor quality of care; and poor healthcare provider performance. Key determinants of coverage include education, knowledge about malaria, socioeconomic determinants, and employment status [52-55].

With the recent increase in attention geared towards community-based delivery and its ever-expanding repertoire of services, systems must be identified to ensure training, support, and incentives for CBIs. Lack of supplies including ITNs and antimalarials have also been reported as a barrier to program success, and hence routine supply of these commodities needs to be guaranteed [16]. Another major barrier to sustainability of such programs is the issue of understaffing at health units [34]. There is also a need to address the challenges of fast growing cities and enhance the health conditions of its inhabitants [17]. Scaling-up environmental activities will require resources for initial massive cleaning and structural repairs that is not possible without donor support [17]. Community involvement remains an essential component of malaria control measures as these interventions require implementation at the household level and the disease is more prevalent in settings with limited access to health facilities. Building community ownership for creating demand for ITNs and increasing trust on CHWs is pivotal for any community-based program to be successful and improve health behaviors.

Interventions such as the distribution of ITNs, net impregnation, IPTp, and IPTc have the potential to be integrated with existing programs such as ANC, immunizations, deworming campaigns and child health days, and could provide useful models for evaluation. The WHO recommends seasonal malaria chemoprophylaxis to be delivered in integration with existing community-based programs, however, a single deployment strategy has not yet been devised [1]. Community case management (CCM) that integrates the management of childhood diarrhea, pneumonia, and malaria is one of the strategies that has received government support and has the potential to improve access. This program utilizes existing CHWs to treat children during home visits and has led to an improvement in ITNs usage and timely malaria treatment for children [56-58]. Similarly, a sharp increase in the number of distributed ITNs have been observed during child health days [59,60]. Many countries in Africa have successfully implemented various integrated models of delivery, however, these are yet to be formally evaluated for effectiveness. A recent analysis carried out in Malawi, Rwanda, Kenya, and Senegal to determine the cost of providing integrated CCM concluded that this is associated with lower costs provided it is used by sufficient numbers of patients to justify the costs of training, equipping, managing, and supervising the CHWs who provide the services [61,62]. However, simultaneous efforts should also be concerted to prevent over diagnosis and drug resistance.

## Conclusion

Collaborative partnerships between governments and donors for the establishment of healthier environments for malaria prevention could play a crucial role in building an ideal platform for malaria specific interventions. Such programs could be articulated in collaborative partnerships between the government and various institutions such as engineering, waste management, education, and public health. We conclude that community-based strategy to deliver malaria specific interventions including ITNs, IRS and IPT, in combination with community education and sanitation, can be effective in reducing the overall burden of malaria morbidity and mortality, especially in malaria endemic areas.

## Abbreviations

ANC: Antenatal care; CBI: Community based intervention; CI: Confidence interval; CQ: Chloroquine; Hb: Hemoglobin; cRCT: Cluster randomized controlled trial; HIC: High-income country; IDoP: Infectious diseases of poverty; IPTc: Intermittent preventive therapy during childhood; IPTp: Intermittent preventive therapy during pregnancy; IRS: Indoor residual spraying; ITN: Insecticide treated net; LMIC: Low- middle- income country; NTD: Neglected tropical disease; PHC: Primary health care; RBM: Roll back malaria; RCT: Randomized controlled trial; RR: Relative risk; SMD: Standard mean difference; SP: Sulfadoxine-pyrimethamine; VHW: Village health worker; WHO: World Health Organization.

## Competing interests

The authors declare that they have no financial or non-financial competing interests.

## Authors’ contributions

ZAB was responsible for designing and coordinating the review. ZSL and RAS were responsible for the data collection, screening of the search results, screening of the retrieved papers against the inclusion criteria, appraising the quality of papers, and abstracting the data. RAS, JKD, and ZSL were responsible for data interpretation and writing the review. ZAB critically reviewed and modified the manuscript. All authors read and approved the final manuscript.

## Supplementary Material

Additional file 1 Multilingual abstracts in the six official working languages of the United Nations. Click here for file
