# Supplementary material for: Impact of community-based interventions for the prevention and control of malaria on intervention coverage and health outcomes for the prevention and control of malaria
Source: Infect Dis Poverty. 2014 Aug 1;3:25. doi: 10.1186/2049-9957-3-25 (PMC4128612; doi:10.1186/2049-9957-3-25)
Supplement: Additional file 1 — Multilingual abstracts in the six official working languages of the United Nations. [file 2049-9957-3-25-S1.pdf]

Translation of the abstract into the six official working languages of the United Nations

تأثير التدخلات المجتمعية الهادفة إلى مكافحة والحد من الملاريا على رقعة انتشار التدخل والنتائج الصحية

ريحانة أ سلام، جاي ك داس، زهرة س لاسي، ذو الفقار أ بوتا

ملخص

توخينا عبر هذه الورقة تقييم فعالية إجراء التخلات المجتمعية من أجل مكافحة وإدارة الملاريا. أجرينا مراجعة ممنهجة وحددنا 42 دراسة لتضمنيها في المراجعة. 25 دراسة من تلك الدراسات شملت دراسات تقييم تأثير التوزيع المجتمعي للناموسيات المعالجة بمبيدات الحشرات (ITN)، رش المنازل بمبيد ذو أثر باقي (IRS)، أو شراشف الأسرة المشبعة بالمبيد. 14 دراسة قامت بتقييم العلاج الوقائي المتقطع (IPT) الموفر عبر أدوات مجتمعية. دراستين ركزتا على التثقيف المجتمعي لمكافحة الملاريا. في حين قامت دراسة واحدة بتقييم الإدارة البيئية عبر تنظيف مجاري الصرف. تشير التحليلات إلى أن إتاحة تدخلات مكافحة وإدارة الملاريا عبر الوسائل المجتمعية بشكل عام أظهرت زيادة ملحوظة في ملكية ITN (RR: 2.16, 95% CI: 1.86, 2.52) واستخدامها (RR: 1.77, 95% CI: 1.48, 2.11). من ناحية أخرى، اقتصر استخدام ITN على ثلثي الأشخاص الذين يملكونها. كما أظهرت أيضا الاستراتيجيات المبنية على المجتمع تناقصا ملحوظا في انتشار طفيليات الدم (RR: 0.56, 95% CI: 0.42, 0.74)، انتشار الملاريا (RR: 0.46, 95% CI: 0.29, 0.73)، حالات الإصابة بالملاريا (RR: 0.70, 95% CI: 0.54, 0.90)، وانتشار فقر الدم (RR: 0.79, 95% CI: 0.64, 0.97). لاحظنا تأثيرا ضعيفا على الإصابة بتضخم الطحال، نتائج الولادة (قلة وزن المولود، الولادة المبكرة، الإملاص/الإجهاض)، القياسات الجسم البشرية (التقزم، الضمور والوزن الناقص)، ومعدل الوفيات (كافة الأسباب والأسباب المتعلقة بالملاريا فقط). هذه النتائج مبنية على عدد محدود من الدراسات التي تم تجميعها بمستوى عال من التغايرية وبالتالي يجب تفسيرها بحذر. تحليل المجموعة الفرعية أشار أن توزيع ITN وشراشف الأسرة المشبعة بالمبيد وIRS عبر الوسائل المجتمعية وإتاحة IPT عبر الوسائل المجتمعية هي استراتيجيات فعالة. الإتاحة عبر الوسائل المجتمعية لمكافحة والحد من الملاريا هي استراتيجيات مؤثرة لتحسين رقعة التغطية والوصول وتقليص خطر الملاريا. من ناحية أخرى، يجب تنسيق الجهود لتفادي التشخيص المفرط ومقاومة الدواء.

Translated from English version into Arabic by Assem Mazloum, through

TRANSLATORS
WITHOUT BORDERS

疟疾预防与控制的社区干预对干预措施覆盖率与健康结局的影响

Rehana A Salam, Jai K Das, Zohra S Lassi, Zulfiqar A Bhutta

摘要

本文旨在评价用于疟疾预防与管理的社区干预的有效性。系统综述了符合要求的42 项研究。其中，25 项评价以社区为单位分发药浸蚊帐、室内滞留喷洒或浸泡床单等措施的影响；14 项评价社区开展的间歇性预防治疗；2 项重点关注疟疾预防的社区健康教育；还有 1 项评价了通过污水治理的环境管理。分析显示，疟疾防控社区干预的实施在药浸蚊帐拥有率（RR: 2.16, 95% CI: 1.86, 2.52）和使用率（RR: 1.77, 95% CI: 1.48, 2.11）越高的地区显著增加。然而，只有 2/3 的药浸蚊帐拥有者使用该类蚊帐。社区干预措施可显著降低原虫血症（RR: 0.56, 95% CI: 0.42, 0.74）、疟疾患病率（RR: 0.46, 95% CI: 0.29, 0.73）、发病率（RR: 0.70, 95% CI: 0.54, 0.90）和贫血率（RR: 0.79, 95% CI: 0.64, 0.97），但对脾肿大、分娩结局（低出生体重、早产、死胎/流产）、人体测量（萎缩、消瘦和体重不足）和死亡率（全死因和疟疾特异性死亡）无显著影响。以上结果是基于有限的研究项目，这些项目存在很高的异质性，因此解读时需要注意。亚组分析认为社区分发药浸蚊帐、浸泡床单、在社区范围进行室内滞留喷洒与间歇性预防治疗等的社区干预措施是有效的。疟疾防控的社区干预措施既能有效提高措施覆盖率和使用水平，又能降低疟疾的疾病负担，并且这些努力应一道来预防疟疾的过度诊断和药物抗性。

Translated from English version into Chinese by Yin Jian-hai, through

Impact des interventions communautaires de prévention et de lutte contre le paludisme sur la couverture d'intervention et les résultats sanitaires

Rehana A Salam, Jai K Das, Zohra S Lassi, Zulfiqar A Bhutta

Résumé

Le présent article évalue l'efficacité des interventions menées au niveau communautaire pour la prévention et la gestion du paludisme. Les auteurs ont procédé à une revue systématique et identifié 42 études à inclure. Vingt-cinq de ces études évaluaient l'impact de la distribution dans la communauté de moustiquaires imprégnées d'insecticide (MII), de nébulisations rémanentes pour l'intérieur (NR) et de draps de lit imprégnés ; quatorze études évaluaient les traitements préventifs intermittents (TPI) dans le cadre communautaire, deux l'éducation communautaire à la prévention du paludisme, et une la gestion de l'environnement (curage de rigoles). L'analyse suggère que les interventions au niveau communautaire visant la prévention et la lutte contre la malaria ont donné, globalement, une augmentation significative de la possession de moustiquaires imprégnées (RR : 2,16, IC 95 % : 1,86, 2,52) et de leur utilisation (RR : 1,77, IC 95 % : 1,48, 2,11). L'utilisation des MII était toutefois limitée aux deux tiers de la population qui en disposaient. Les stratégies communautaires ont également donné une diminution significative de la parasitémie (RR : 0,56, IC 95 % : 0,42, 0,74), de la prévalence du paludisme (RR : 0,46, IC 95 % : 0,29, 0,73), de l'incidence du paludisme (RR : 0,70, IC 95 % : 0,54, 0,90) et de la prévalence de l'anémie (RR : 0,79, IC 95 % : 0,64, 0,97). Nous avons constaté un impact non significatif sur la splénomégalie, les paramètres néonataux (faible poids de naissance, prématurité, mortinatalité/fausses couches), les mesures anthropométriques (retard staturopondéral, fonte de la masse musculaire et faible poids) et la mortalité (par toutes causes et spécifiquement liée au paludisme). Ces observations se basent sur l'évaluation groupée d'un nombre limité d'études très hétérogènes et doivent donc être interprétées avec prudence. L'analyse par sous-groupes suggère que la distribution communautaire de moustiquaires et draps de lit imprégnés et de produits de nébulisation rémanente ainsi que et le déploiement communautaire de TPI sont des stratégies efficaces. La réalisation des interventions de prévention et de lutte contre le paludisme au niveau communautaire constitue une stratégie efficace pour améliorer la couverture et l'accès et réduire le poids du paludisme. Il importe cependant aussi de concerter les efforts afin d'éviter surdiagnostics et résistances médicamenteuses.

Translated from English version into French by Suzanne Assenat, through

TRANSLATORS
WITHOUT BORDERS

Воздействие мер по предотвращению и контролю распространения малярии на базе сообщества на конечный охват программы и здоровье населения

Риана А. Салам, Джай К. Дас, Зохра С. Ласси, Зульфикар А. Бхутта

Резюме

Целью данной работы являлась оценка эффективности реализации мер по предотвращению и контролю распространения малярии на базе сообщества. Мы провели систематический анализ и выявили 42 исследования, подлежащих изучению. В рамках двадцати пяти из таких исследований проводилась оценка воздействия распространения инсектицидных сеток, распыления инсектицидов остаточного действия в помещениях или пропитки постельного белья на базе сообщества; четырнадцать исследований оценивали периодическое превентивное лечение, проводящееся внутри сообществ; два исследования были направлены на изучение просветительских мер, направленных на предотвращение малярии, на базе сообщества; в то время как одно исследование содержало оценку экологического управления за счет очистки стоков. Согласно результатам анализа, повсеместная реализация мер на базе сообщества, направленных на предотвращение и контроль малярии, свидетельствует о значительном росте числа инсектицидных сеток (RR: 2.16, 95% CI: 1.86, 2.52) и интенсивности их использования (RR: 1.77, 95% CI: 1.48, 2.11). Однако использование инсектицидных сеток было ограничено двумя третями населения, которые могли прибрести их. Стратегии, реализуемые на базе сообщества, также продемонстрировали значительное сокращение случаев наличия паразитов в крови (RR: 0.56, 95% CI: 0.42, 0.74), распространения малярии (RR: 0.46, 95% CI: 0.29, 0.73), числа заболевших малярией (RR: 0.70, 95% CI: 0.54, 0.90) и распространения анемии (RR: 0.79, 95% CI: 0.64, 0.97). Мы обнаружили незначительное воздействие на спленомегалию, показатели рождаемости (низкий вес новорожденных, преждевременные роды, замершая беременность/выкидыш), антропометрические показатели (низкий рост, упадок сил и дефицит веса) и уровень смертности (по всем причинам, включая малярию). Эти результаты основываются на ограниченном числе проведенных исследований с высоким уровнем разнородности, так что интерпретировать их следует с осторожностью. Анализ подгрупп подтвердил тот факт, что

распространение инсектицидных сеток, пропитанного постельного белья и распыление инсектицидов остаточного действия, а также периодическое превентивное лечение на базе сообщества являются эффективными стратегиями по улучшению охвата и доступа, а также по сокращению случаев заболеваемости малярией; однако также необходимо сосредоточить усилия на борьбе с постановкой слишком большого количества диагнозов и устойчивостью к медикаментам.

Translated from English version into Russian by Irina Zayonchkovskaya, through

TRANSLATORS
WITHOUT BORDERS

Impacto de Intervenciones Basadas en la Comunidad para la Prevención y el Control de la Malaria en Cobertura de Intervención y Resultados de Salud

Rehana A Salam, Jai K Das, Zohra S Lassi, Zulfiqar A Bhutta

Abstracto

En este documento, pretendimos evaluar la efectividad de la entrega de intervenciones basadas en la comunidad para la prevención y la administración de malaria. Realizamos una revisión sistemática e identificamos 42 estudios para inclusión. Veinticinco de los estudios incluidos evaluaron el impacto de la distribución basada en la comunidad de redes tratadas con insecticidas (*insenticide treated nets* – ITN), rociados residuales de interiores *(indoor residual spraying* – IRS), o sábanas impregnadas; catorce estudios evaluaron la terapia preventiva intermitente *(intermittent preventive therapy* – IPT) ofrecida en ambientes comunitarios; dos estudios se concentraron en la educación basada en la comunidad para la prevención de la malaria; mientras que un estudio evaluó la gestión ambiental a través del lavado de drenajes. Los análisis sugieren que, en general, la entrega de intervenciones basadas en la comunidad para prevenir y controlar la malaria mostraron un significativo incremento en propiedad de ITN (RR: 2.16, 95% CI: 1.86, 2.52) y uso (RR: 1.77, 95% CI: 1.48, 2.11). Sin embargo, el uso de ITN se limitó a dos tercios de la población que las tenía. Las estrategias basadas en la comunidad también mostraron una significativa disminución en parasitemia (RR: 0.56, 95% CI: 0.42, 0.74), prevalencia de malaria (RR: 0.46, 95% CI: 0.29, 0.73), incidencia de malaria (RR: 0.70, 95% CI: 0.54, 0.90) y prevalencia de anemia (RR: 0.79, 95% CI: 0.64, 0.97). Encontramos un impacto no-significativo sobre la esplenomegalia, resultados de nacimientos (bajo peso de nacimiento, prematuridad, abortos/muertes fetales), medidas antropométricas (retraso del crecimiento, desgaste y bajo peso) y mortalidad (por cualquier causa y específicas de la malaria). Estos hallazgos están basados en un número limitado de estudios reunidos con un alto nivel de heterogeneidad y por ello deben ser interpretados con precaución. El análisis de sub-grupos sugirió que la distribución basada en la comunidad de ITNs, sábanas impregnadas e IRS, y la entrega basada en la comunidad de IPT son estrategias efectivas. La entrega de intervenciones basadas en la comunidad para prevenir y controlar la malaria son estrategias efectivas para mejorar la cobertura y el acceso y para reducir la carga de

malaria; sin embargo, también se debe concertar esfuerzos para evitar el sobre-diagnóstico y la resistencia a las drogas.

Translated from English version into Spanish by Denise Tarud, through

TRANSLATORS
WITHOUT BORDERS
